# Nerve ultrasound, neuronopathy and cough predict sensory neuropathy patients with *RFC1* expansions

**DOI:** 10.1093/braincomms/fcaf434

**Published:** 2025-11-03

**Authors:** Anthony Garvey, I Zay Melville, Carolin K Scriba, Vivien Yong, Miriam Rodrigues, Justin Kao, Melanie Glenn, Shilpan Patel, Thomas Chang, James Caldwell, Caitlyn Ren, Nigel G Laing, Gianina Ravenscroft, Luciana Pelosi, Rachael L Taylor, Richard Roxburgh

**Affiliations:** Neurology Department, Dunedin Hospital, He hauora, he kuru pounamu, Health New Zealand, Te Whatu Ora, Dunedin 9054, New Zealand; Physiology Department, Centre for Brain Research, University of Auckland, Waipapa Taumata Rau, Auckland 1023, New Zealand; Neurogenetic Diseases Group, Centre for Medical Research, QEII Medical Centre, University of Western Australia, Nedlands, WA 6009, Australia; Neurology Department, Tauranga Hospital, Health New Zealand, Te Whatu Ora, Tauranga 3112, New Zealand; Neurology Department, Auckland City Hospital, Te Toka Tumai, Health New Zealand, Te Whatu Ora, Auckland 1023, New Zealand; Radiology Department, Auckland City Hospital, Te Toka Tumai, Health New Zealand, Te Whatu Ora, Auckland 1023, New Zealand; Neurology Department, Auckland City Hospital, Te Toka Tumai, Health New Zealand, Te Whatu Ora, Auckland 1023, New Zealand; Neurology Department, Auckland City Hospital, Te Toka Tumai, Health New Zealand, Te Whatu Ora, Auckland 1023, New Zealand; Radiology Department, Auckland City Hospital, Te Toka Tumai, Health New Zealand, Te Whatu Ora, Auckland 1023, New Zealand; Neurology Department, Auckland City Hospital, Te Toka Tumai, Health New Zealand, Te Whatu Ora, Auckland 1023, New Zealand; Neurology Department, Auckland City Hospital, Te Toka Tumai, Health New Zealand, Te Whatu Ora, Auckland 1023, New Zealand; Radiology Department, Auckland City Hospital, Te Toka Tumai, Health New Zealand, Te Whatu Ora, Auckland 1023, New Zealand; Neurology Department, Auckland City Hospital, Te Toka Tumai, Health New Zealand, Te Whatu Ora, Auckland 1023, New Zealand; Neurogenetic Diseases Group, Centre for Medical Research, QEII Medical Centre, University of Western Australia, Nedlands, WA 6009, Australia; Neurogenetic Diseases Group, Centre for Medical Research, QEII Medical Centre, University of Western Australia, Nedlands, WA 6009, Australia; Neurology Department, Tauranga Hospital, Health New Zealand, Te Whatu Ora, Tauranga 3112, New Zealand; Neurogenetics Clinic, Department of Medicine, Centre for Brain Research, University of Auckland, Waipapa Taumata Rau, Auckland 1023, New Zealand; Physiology Department, Centre for Brain Research, University of Auckland, Waipapa Taumata Rau, Auckland 1023, New Zealand; Neurology Department, Auckland City Hospital, Te Toka Tumai, Health New Zealand, Te Whatu Ora, Auckland 1023, New Zealand; Radiology Department, Auckland City Hospital, Te Toka Tumai, Health New Zealand, Te Whatu Ora, Auckland 1023, New Zealand

**Keywords:** CANVAS syndrome, ataxia, neuropathy, neurogenetics

## Abstract

The finding of biallelic pathogenic pentanucleotide *RFC1* expansions has extended the spectrum of disease in cerebellar ataxia, neuropathy and vestibular areflexia syndrome. It is clear that for many, a sensory neuropathy is an early feature and raises the question of how to identify which patients with this common neurophysiological presentation should be tested genetically for the condition. We identified patients with idiopathic, sensory predominant neuropathies who had attended the Neurophysiology Department of Auckland Hospital for nerve conduction studies. We undertook a systematic clinical re-evaluation to test whether any of the following hypothesized variables distinguish the presence of pathogenic *RFC1* expansions. These were (i) chronic cough, (ii) ataxia, (iii) pure sensory changes on nerve conduction, (iv) a non-length-dependent pattern of sensory loss on nerve conduction studies, (v) small nerves on peripheral nerve ultrasound, (vi) bilateral vestibular dysfunction and (vii) autonomic dysfunction. We recruited 53 patients, of whom 10 had normal repeat nerve conductions. Among the 43 (25 males, 18 females) remaining patients, five were positive for the pathogenic *RFC1* expansions. All five reported a chronic cough (versus 4/38 *RFC1*-negative cases, *P* = 0.0002). None of the five cases had abnormal motor findings (versus 20/37 *RFC1*-negative cases, *P* = 0.07). Four of the five cases had small (<5.2 mm^2^) mean upper limb nerves by cross-sectional area on ultrasound (versus 2/38 *RFC1*-negative cases, *P* = 0.0006). The fifth had concurrent diabetes, which might explain their normal sized nerves. Four of the five cases had a non-length-dependent sensory neuropathy, and one had a length-dependent sensory neuropathy. The *RFC1*-positive case with a length-dependent neuropathy had small upper limb nerves on ultrasound. There were no differences in ataxia scores between the groups, and only two *RFC1*-positive cases had vestibular involvement. Two of the five *RFC1*-positive cases, both Sāmoan, had a novel arrangement in their *RFC1* expansion in which the pathogenic AAGGG expansion was preceded by a short AAAAG expansion. Taken together, in this small sample, the presence of a chronic cough with either a non-length-dependent neuropathy on nerve conduction studies or a mean upper limb nerve cross-sectional area <5.2 mm^2^ was strongly associated with the *RFC1* expansion (sensitivity 100%, specificity 97%). Patients who fit these criteria should be tested genetically for *RFC1*. Ultrasound and nerve conduction studies should be seen as complementary in the workup of patients for *RFC1* expansions.

## Teaser

Garvey *et al.* report that in a cohort of patients with unexplained, sensory predominant axonal neuropathies, the presence of chronic cough with either small nerves on ultrasound, or non-length-dependent neuropathy was strongly associated with the *RFC1* gene expansion found in CANVAS (cerebellar ataxia, neuropathy and vestibular areflexia syndrome).

## Graphical Abstract

**Graphical Abstract:**
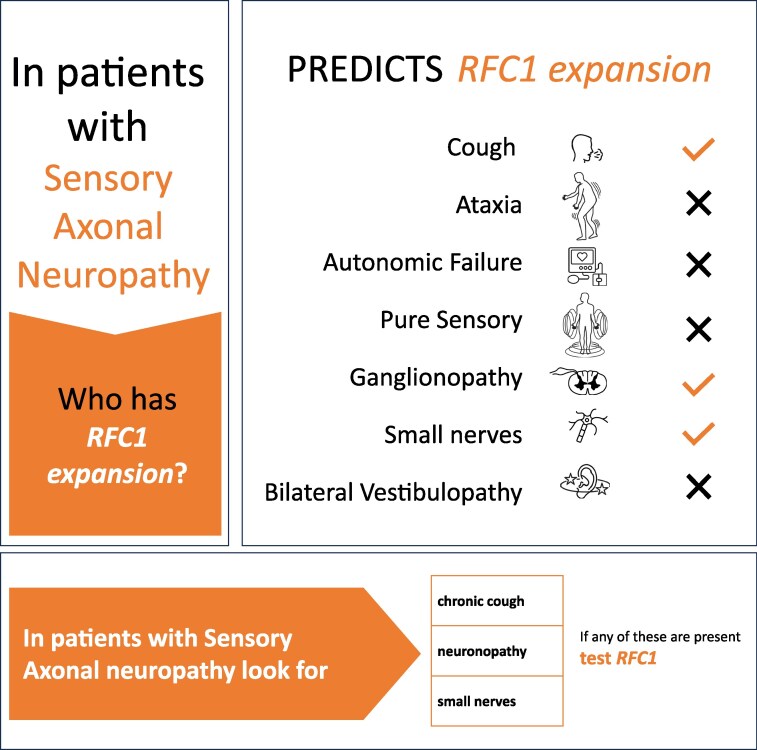

## Introduction

The clinical triad of cerebellar ataxia, neuropathy and vestibular areflexia syndrome (CANVAS) was first described by Bronstein *et al*.^1^ in 1991. In 2004, a cohort of patients with bilateral vestibulopathy and progressive ataxia was described, three of whom also had an ‘axonal sensorimotor neuropathy’.^2^ In 2011, Szmulewicz *et al.*^3^ named the syndrome CANVAS and described a predominantly sensory and length-dependent neuropathy in their cohort; they also demonstrated that the pathological correlate of this was a dorsal root ganglionopathy.^4^ Subsequently, autonomic involvement and chronic cough,^5^ and small peripheral nerves on ultrasound were described.^6^ The most significant advance came with the discovery in 2019 of pathological pentanucleotide expansions in intron 2 of the *RFC1* gene as the cause of the syndrome.^7^

Chronic cough has been confirmed to be a common feature in genetically confirmed patients, present in 64 of 100 patients in one study.^8^ Various research teams have shown that the expansion explains a small proportion (∼3%) of non-selected ataxia patients^9,10^ and higher proportions (∼15%) of late-onset disease.^11-13^ It is also found in patients with downbeat nystagmus,^14^ and with isolated bilateral vestibulopathy.^15^

Sensory neuropathy appears to be a core feature of the condition,^9,16,17^ leading many to regard ‘RFC1 spectrum disorder’ as a ‘neuropathy plus’ rather than an ‘ataxia plus’ syndrome. In 2021, two studies explored the role of *RFC1* in axonal neuropathy.^18,19^ Curro *et al.*^18^ evaluated 225 patients with a sensory-predominant axonal neuropathy on nerve conduction studies (NCS) for the *RFC1* expansion. None of the 100 patients with significant motor involvement had an *RFC1* expansion, but 34% of the 125 with a pure sensory axonal neuropathy did. Of these, 70% had cough, 50% had cerebellar signs and 46% had bilateral vestibulopathy. However, few patients in this study had formal vestibular testing. Tagliapietra *et al.*^19^ reviewed the notes and investigations of 234 patients who had been worked up for sural nerve biopsy and had DNA available. Two per cent of 138 patients with sensorimotor, 18% of 56 patients with sensory predominant and 53% of 40 patients with pure sensory neuropathy had bi-allelic *RFC1* repeat expansions. Gene-positive patients were more likely to have chronic cough (9% versus 0), absent sural SNAPs and retained reflexes. Only one (*RFC1* negative) patient had vestibular involvement. However, clinical assessments were neither thorough (e.g. very low rates of formal vestibular function testing) nor systematic (e.g. only 37 patients in the first study were asked about cough), which makes it hard to draw conclusions about how common and distinguishing these non-neuropathy features are.

Such high rates of genetic positivity could justify testing all patients with sensory axonal neuropathy for *RFC1* expansion, but as this is a common finding on NCS, this would be expensive. We, therefore, undertook a prospective systematic study to determine what aspects of patients’ clinical presentation and investigations could predict a positive result and justify genetic testing. We employed systematic history, examination and investigations to test seven primary hypotheses regarding features whose presence might distinguish *RFC1* expansion patients: chronic cough; ataxia; pure sensory changes on NCS; non-length-dependent pattern of sensory loss on NCS; small nerves on peripheral nerve ultrasound; bilateral vestibular dysfunction; and autonomic dysfunction.

## Materials and methods

### Primary end-points

We tested the seven hypotheses by assessing seven primary end-points using established clinical scores and assessments (Table 1). Chronic cough was assessed on history; if present, participants were invited to rate their cough on a visual analogue scale. Ataxia was assessed using the Scale for the Assessment and Rating of Ataxia (SARA) score.^20^ Motor involvement was defined as any abnormal upper limb (UL) motor amplitude or a reduction of more than 50% in a lower limb motor amplitude on repeat NCS. The presence of non-length-dependent neuropathy was adjudged using a modification of the SNAP (sensory nerve amplitude potential) asymmetry score,^21^ which we assessed on a prospectively repeated NCS. We adapted the score by including an alternative criterion of bilateral absence of SNAPs, in addition to the existing criterion of > 50% side-to-side amplitude asymmetry, stipulating that one of these had to be present in three nerve pairs for that person to be considered as having a non-length-dependent neuropathy. We justify this because bilateral absence is a feature of more severe neuronopathy, as the authors acknowledge.^21^ Nerve size was evaluated using the mean UL nerve cross-sectional area (mean UL CSA), which we have shown in previous studies to be a sensitive and practical measure.^6,22,23^ Vestibular function was assessed using the gain on the better side (as we were looking for bilateral vestibulopathy) on horizontal video head impulse test. Autonomic dysfunction was measured following Ewing and Clarke.^24^ In addition to these primary end-points, we undertook extensive evaluations of the patients, including alternate methods for assessing our primary end-points and testing secondary hypotheses (Table 1). Importantly, our team members performed their examinations and investigations blinded to each other’s findings and the genetic testing results.

**Table 1:** Primary and secondary end-points

Primary hypothesis variable	Primary end-point	Secondary end-point
Cough	Yes/no (history)	
Ataxia	SARA score (exam)	
Pure sensory (versus sensorimotor)	Yes/no (repeat NCS)	
Neuronopathy	Modified SNAP asymmetry score (NCS)	Non-acral sensory loss (exam) Ulnar sensory-motor amplitude ratio (NCS) Mean UL SNAPs (NCS) Camdessanché score (history and NCS)
Nerve size	Mean UL CSA (US)	
Vestibular function	Horizontal vHIT (gain on better side)	Bedside VOR Bedside VVOR Refixation (catch up) saccades on vHIT Video VVOR gain
Autonomic function	Formal autonomic testing (Ewing protocol)	SAS—number of symptoms SAS—severity of symptoms
**Other end-points**		
Severity of Sensory involvement		ISS TCNS
Presence of positive sensory symptoms/pain		History of positive symptoms/pain

Abbreviations: CSA, cross sectional area; NCS, nerve conduction studies; SNAP, sensory nerve action potentials; SAS, Survey of Autonomic Symptoms. ISS, INCAT Sensory Sumscore; TCNS, Toronto Clinical Neuropathy Score; UL, upper limb; US, nerve ultrasound; vHIT, video head impulse test.

### Consent and ethics

The study was performed in accordance with the Declaration of Helsinki; all participants gave written consent. Our Genetic Counsellor explained the implications of genetic tests. Ethics was granted through the New Zealand Health and Disability Ethics Committee (HDEC 2023 10513) and included specific permission for the storing and analysis of DNA within and outside New Zealand.

### Participant screening and clinical assessments

The records of the Auckland Hospital neurophysiology department between January 2016 and July 2023 were reviewed. Orthopaedic referrals were excluded, being almost exclusively entrapment neuropathies and radiculopathies queries.

Reports were screened; those reporting a pure sensory, or sensory predominant axonal neuropathy were included. By our definition, this was at least two sensory nerves with a sensory nerve action potential <50% of the lower limit of normal and <50% reduction in the tibial motor amplitude, only.

Participants with an entrapment neuropathy or a clear cause of their condition in their medical records, such as diabetes, alcoholism or auto-immune disease, were excluded, as were patients previously tested for *RFC1*.

### Neurological history and examination

After recruitment, participants were asked about any conditions that might cause a secondary acquired neuropathy, such as diabetes, cancer and excessive alcohol intake. We specifically asked about positive sensory symptoms such as tingling and pain, and reviewed the patients’ medical records for age of onset and presenting symptoms. To look for evidence of previous ataxia, we reviewed whether patients had had MRI of the brain and their indication. We had all scans reviewed for signs of cerebellar atrophy by an experienced neuroradiologist blinded to the genetic diagnosis.

The patients then underwent a comprehensive neurological examination. This included the specific elements of SARA score, the Inflammatory Neuropathy Cause and Treatment (INCAT) Sensory Sumscore (ISS) and the Toronto Clinical Neuropathy Score (TCNS), as well as eye movements and bedside vestibular function using the head impulse test,^25,26^ and visually enhanced vestibulo-ocular reflex (VVOR).^27^

### Repeat nerve conduction testing

All patients underwent repeat NCS (Natus, Nicolet). This included bilateral antidromic median and ulnar sensory responses from digits II & V, respectively, the superficial radial sensory and sural sensory nerves, as well as median, ulnar and tibial motor responses. This also allowed us to assess secondary end-points such as ulnar sensory-motor amplitude ratio^28^ and the mean of the UL sensory nerve action potentials (mean UL SNAPs) and the Camdessanché score,^29^ a widely used predictor of neuronopathy. The lower limit of normal for UL sensory nerve action potentials was 10 uV, and, for the sural nerve, 4 uV. The lower limit of normal for the median motor amplitude was 4.5 mV, for the ulnar nerve, 6.0 mV, and for the tibial nerve, 4.0 mV.

### Nerve ultrasound

Nerve ultrasound was performed using the SonoSite XPort system (Fujifilm SonoSite Inc., Bothel, WA, USA) equipped with a 6–15 MHz linear array transducer. The median and ulnar nerves were scanned on the patient’s dominant side from axilla to wrist. The nerve cross-sectional areas (CSAs) were measured at mid-forearm and mid-humerus with manual tracing inside the hyperechoic rim of the epineurium. Three measurements were made at each site, and the mean value was taken.

The CSAs of the individual patients were compared with our laboratory reference values from 43 healthy controls. The normal ranges (mean ± 2 SD in normal controls) are: median nerve, >4.0 and <8.0 mm^2^ at mid-forearm, and >5.6 and <12.0 mm^2^ at mid-humerus; ulnar nerve, >3.8 and <7.8 mm^2^ at mid-forearm, and >3.7 and <7.7 mm^2^ at mid-humerus, and mean UL CSA, >4.9 and <8.1 mm^2^.

### Video oculography

We undertook horizontal video head impulse testing (vHIT) and video VVOR (vVVOR). The ICS Impulse (Natus, Taastrup, Denmark) video-oculography device simultaneously recorded horizontal head and eye movements. For both tests, the participant sat in a straight-backed chair while fixating on a stationary target positioned on a wall 130 cm in front of them. Outcome measures for vHIT included the vestibulo-ocular reflex (VOR) gain, defined as the mean ratio of the area under each eye and head velocity curve, and the offline calculation of rates of refixation (catch-up) saccades. Results were classified with reference to Bárány Society criteria for bilateral vestibulopathy (bilateral gain < 0.6)^30^ as well as laboratory normative data, which considers both gain and catch-up saccades by age category.^31^ Catch-up saccade rate was defined as the number of saccades with velocity >100°/s divided by the number of head impulses performed in a given direction. VVOR testing was performed with the examiner moving the participant’s head side-to-side in a sinusoidal motion from behind, while the participant fixated on the stationary target. The examiner rotated the participant’s head at 0.5 Hz, controlling head rotation via a velocity trace and a metronome (only audible to the examiner).

Before analysis, the data were manually examined for any disruptive artefacts. VVOR data were analysed using MATLAB.^32^ The programme determined gain values using an area under the curve (AUC) method. Results were compared with laboratory normative data [abnormal vVVOR <0.91 (right) and 0.86 (left)].

### Autonomic nervous system testing and symptoms assessment

Following Ewing and Clarke,^24^ sympathetic function was assessed by blood pressure response to postural change and sustained grip; parasympathetic function by recording RR interval variation in response to postural change, deep breathing and Valsalva manoeuvre. Autonomic symptoms were assessed using the Survey of Autonomic Symptoms,^33^ which rates patients on the presence and severity autonomic severity score of 12 (women 11) autonomic symptoms.

### Blood tests for confounding conditions

All patients had a new workup for baseline bloods; vitamin B_12_, thyroid-stimulating hormone, HbA1c, anti-nuclear antibodies and serum electrophoresis. For genetic testing, DNA was extracted from a 10 ml ethylenediaminetetraacetic acid (EDTA) blood sample using standard methods. An initial deidentified sample was sent to the *Harry Perkins Institute of Medical Research* in Perth, Western Australia, for *RFC1* testing using a two stage repeat primed PCR methodology as previously described.^13^ Positive results were then verified through the clinical pathway from Canterbury Health Laboratories to Pathwest Laboratories in Perth, Western Australia.

### Statistical analysis

Descriptive statistics were used to characterize our cohort. When describing the whole group, we report the mean (±standard deviation). Since the group of *RFC1*-positive patients was small, for quantitative data, we have reported median (lower quartile, upper quartile). When comparing *RFC1*-positive and *RFC1*-negative groups, we have used Fisher’s exact test for categorical data and Mann–Whitney U-test for quantitative data. Receiver operator curve (ROC) analyses were performed for continuous variables, which showed evidence of being predictive of *RFC1* expansion. We also calculated sensitivity, specificity and Youden index values to identify the best predictors of *RFC1* positivity. Figures for summary data were prepared using Microsoft Excel or SPSS (version 29) and formatted using Adobe Illustrator (version 16).

An alpha level of 0.05 determined statistical significance. With respect to multiple testing, although we recorded a large number of clinical parameters, we really set out to explore seven hypotheses—but ended up only testing six. We used the Holm–Bonferroni method^34^ to correct for multiple testing for these six hypotheses. Other nominally significant secondary measures should be regarded as exploratory.

## Results

**Figure 1:**
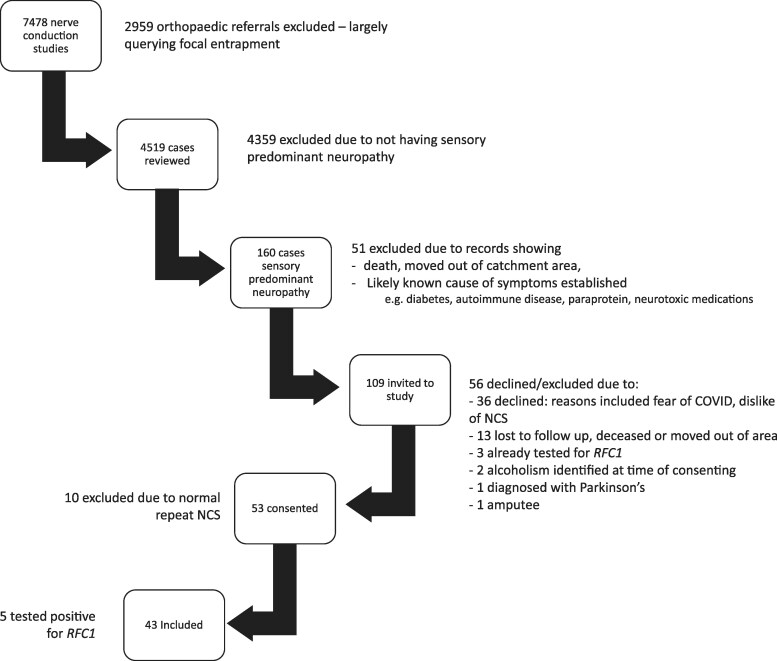
Flowchart of patient screening.

Between 2015 and 2023, 7478 NCS were performed. In total, 2959 orthopaedic referrals were not reviewed (Fig. 1). Of the 4519 remaining cases, 160 had a sensory predominant neuropathy. Fifty-one were excluded as their records showed that they had died, moved out of area or had an alternative diagnosis. One hundred and nine patients were approached; 36 declined, 13 had died, couldn’t be contacted or had moved out of area. Three had already been tested for *RFC1*; four were excluded due to diagnoses of Parkinson’s disease, alcoholism and bilateral amputations. This left 53 who agreed to participate in the study and attended the clinical assessments. Of note, two of the three who had had previous testing for *RFC1* were positive—both had had their NCS requested for investigation of ataxia. One had a non-length-dependent sensory neuropathy and the other a length-dependent sensory neuropathy. Neither had had nerve ultrasound.

Ten of the 53 patients had normal NCS on repeat testing. On reviewing their original studies, two had low amplitude medial plantar responses, which were not used in our study as it is often not recordable in healthy individuals over age 55. In one patient, the diagnosis of peripheral neuropathy was based on mild slowing of sensory conduction velocities, which may have been temperature-related. In the remaining six patients, the sole abnormality was low amplitude sural sensory responses (three below reference range and two deemed low for the patient’s age). These 10 patients were removed from further analysis.

Of the 43 remaining patients, 25 were male, 18 female. 37 identified as New Zealanders of European ancestry or as European, three Sāmoan, two Indian and one Māori. The average age was 62 (range 26–80).

### Medical history and neurological examination

All 43 patients attended the neurological exam and history appointments. Symptom onset was 54.7 (±10.5) years. No additional participants reported a history of diabetes or autoimmune disease. Six participants had a history of cancer: two with prostate cancer treated with hormone therapy, one with squamous cell carcinoma of the tongue treated with local radiotherapy and surgery, one with bowel cancer treated with surgery alone, one with testicular cancer treated with bleomycin, etoposide and platinum chemotherapy and one patient was diagnosed with lymphoma during the study.

Alcohol consumption was available for 38 of the 43 participants (81%). No patient currently has excessive daily intake. However, six participants described periods in their lives with increased alcohol consumption, sufficient to cause neuropathy, though mostly in the remote past.

Forty-one of the 43 participants gave responses regarding symptoms of cough (95%). Nine (21%) reported having a chronic or persistent cough, scoring from 24 to 100 on the cough visual analogue scale; the mean rating was 53.8 ± 22.7.

All but one patient had sensory symptoms in their feet, most commonly numbness (35/41), followed by paraesthesia (31/41); fewer patients reported weakness (21/41). Of the 35 patients who reported numbness, this was asymmetrical in 16 and symmetrical in 18 (one patient had a below knee amputation). Pain was reported in 22 of the 53 referral letters; onset of symptoms was non-acral in eight patients and asymmetrical in nine.

Few participants had abnormal eye movements. One patient had limited upgaze. Two had horizontal gaze-evoked nystagmus. One had broken horizontal and vertical pursuit, and a second had broken vertical pursuit. Saccade initiation and latency were normal in all patients. One patient had slowed vertical saccades, three had hypometric vertical saccades and one had hypermetric horizontal and vertical saccades.

Bedside vestibular function testing (horizontal head impulse test and VVOR) was completed in 35 of the 43 patients. Bedside horizontal VVOR was abnormal in four patients, and head impulse tests were bilaterally abnormal in six. Five patients had more than one eye motility or vestibular test abnormality. One patient with gaze-evoked nystagmus had bilateral positive head impulse tests. Two of the patients with hypometric vertical saccades had abnormal VVOR, and the other had positive head impulse tests. The patient with slow vertical saccades had positive head impulse tests.

Ataxia was not common—either in the history or on our prospective examination. Nine of the 43 patients had had MRI scans, and only for one of these patients was the indication ‘imbalance’ (others were for headache, possible seizure, asymmetrical hearing loss): SARA score range was 0–10 (out of 40), with mean score of 2.9 ± 2.6 and median of 3.0 (0.5, 4.5). Neuropathy signs were more prominent with the ISS (based on vibration, pinprick and two-point discrimination), ranging from 2 to 15 (out of 20); however, the mean was low at 5.8 ± 3.2 with a median of 5.0 (3.5, 8.0). TCNS, which derives points from a wider range of physical signs, ranged in value from 1 to 13 out of a possible 13. The mean was 6.6 ± 3.1; median 6.0 (4.5, 9.0).

### Nerve conduction results

All 43 patients had repeat NCS. However, one (*RFC1* negative) patient did not tolerate the procedure (a normal right median nerve SNAP had been recorded). Twenty-five (60%) had no motor involvement; of these, five had absent or very low SNAPs, satisfying our modified SNAP asymmetry score criteria for non-length-dependent sensory neuronopathy, and 20 had a length-dependent sensory neuropathy. Seventeen (40%) had motor involvement; four of these also satisfied the modified SNAP asymmetry score criteria for a non-length-dependent pattern and 13 had a sensory-predominant length-dependent neuropathy.

### Nerve ultrasound

Nerve ultrasound was performed on 40 of the 43 participants. Mean UL CSA was reduced in six participants, normal in 18 and increased in 16. The average mean UL CSA was 7.6 ± 2.5 mm^2^, range 3.2–15.1 mm^2^.

### Autonomic symptoms and testing

Forty-two of the 43 patients (98%) completed the Survey of Autonomic Symptoms. The average number of symptoms was 3.4 ± 2.2; median 3 (2, 5); range 0–9 (max 11 for women, 12 for men). The average total score was 11.5 ± 9.1, range 0–38. Of the 43 included patients, 12 were unable to complete autonomic testing due to technical issues with performing or interpreting the test (6/12), medical issues (2/12), or scheduling issues (4/12). Of the 31 patients who did complete testing, just three were completely normal, eight demonstrated signs of mild parasympathetic dysfunction and eight had moderate parasympathetic dysfunction; seven participants had sympathetic dysfunction, all of whom had co-existent parasympathetic involvement; five had sympathetic dysfunction, much greater than parasympathetic dysfunction.

### Vestibular testing

Horizontal vHIT results were available for all 43 patients. Gains on the better side ranged from 0.52 to 1.26 with a mean of 0.98 (±0.14). Four patients had bilaterally reduced gains, two fulfilling Bárány Society criteria for bilateral vestibulopathy (gains < 0.6). A further five had unilateral impairment, two to the right and three to the left. Rates of catch-up saccades ranged from 0 to 1.82. They were bilaterally high in eight patients and unilaterally high in five patients. All but one patient with unilaterally or bilaterally low gains had abnormally high rates of catch-up saccades. Horizontal VVOR results were available in 34 of 43 patients. Gains on the better side ranged from 0.93 to 1.19 with a mean of 1.06 ± 0.06. None of the patients in the cohort had bilaterally reduced VVOR gains.

### Blood tests

All 43 patients underwent a panel of tests for secondary causes of neuropathy. Two patients had low B_12_ levels. No patients had elevated TSH levels. Three patients were found to have developed diabetes in the interval between initial and repeat assessment based on HbA_1c_ testing (56, 46 and 45 mmol/mol, normal range 20–40 mmol/mol), with another eight having borderline results (40–42 mmol/mol). Four had monoclonal gammopathy. Thirteen patients had positive ANAs; 10 at 1:160 or less, one at 1:320 and two at 1:1280—none of these three had dsDNA or ENA antibodies on later clinical testing.

### *RFC1* genetic results

Of the 43 participants, five were found to have bi-allelic pathogenic *RFC1* expansions. Three participants were homozygous for (AAGGG)_exp_ commonly described in European populations.^7^ One participant was homozygous for a novel expanded allele: [AAAAG]_exp_[AAGGG]_exp_, where the pathogenic [AAGGG] expansion was preceded by an AAAAG expansion. The fifth patient carried this same allele in compound heterozygosity with the [AAAGG]_exp_[AAGGG]_exp_ pathogenic expansion first described in Māori and Cook Islands Māori.^35^ Both these patients were Sāmoan.

### Comparison of clinical features between *RFC1* gene-positive and *RFC1* gene-negative patients (Table 2, Fig. 2)

*RFC1* pathological expansion patients ranged in age from 56 to 67 years old [median 62 (62, 64)] at the time of assessment. Three were female, two male. Two were Sāmoan; the other three, European. Median age of onset of symptoms was 53 (49, 58).

#### Medical history and exam findings

Chronic cough was the only feature on history or exam present in all five *RFC1-*positive participants, compared with four of the 38 *RFC1-*negative participants (*P* = 0.0002). Mean cough visual analogue scores in those who reported chronic cough were not different: 52.8 versus 43.0 (*P* = 0.63). No *RFC1*-positive patients had a history of cancer or had a history of heavy alcohol use. Asymmetry of numbness on the questionnaire was nominally significant, with all five *RFC1-*positive patients reporting this compared with 11 of 29 *RFC1-*negative patients *P* = 0.019.

**Figure 2:**
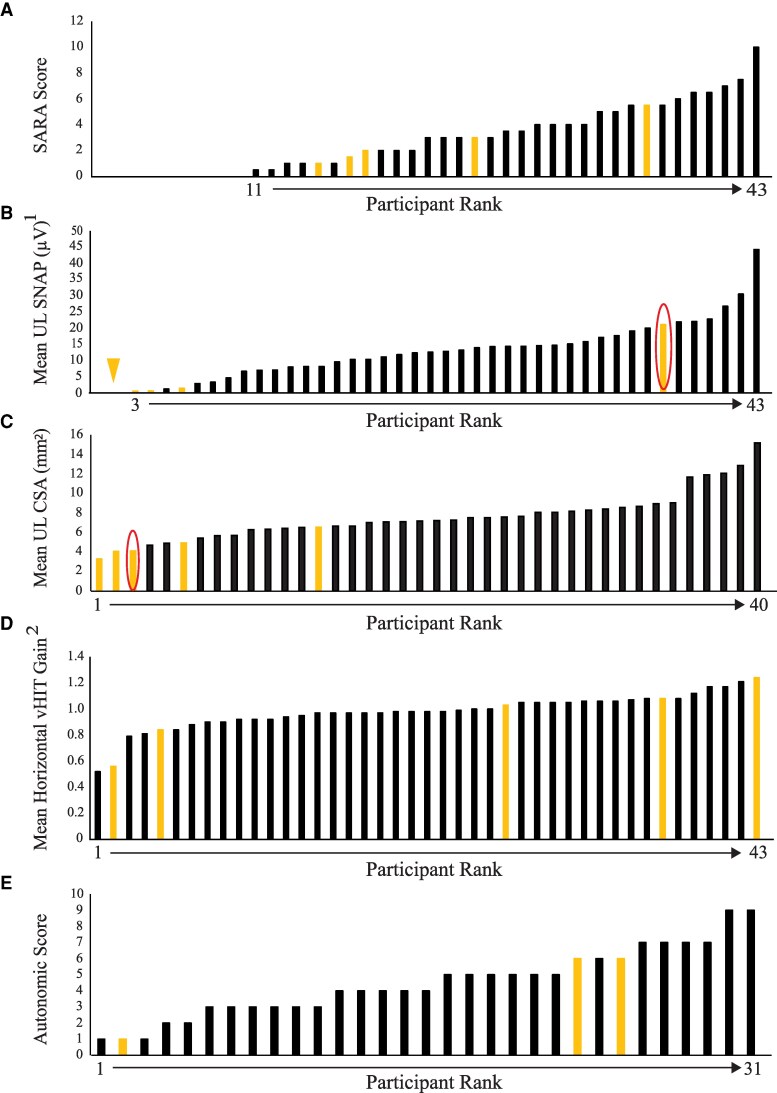
**Ranking of patients according to primary outcome measures.** (**A**) Ataxia (SARA score); (**B**) neuronopathy (mean UL SNAPs); (**C**) nerve size (mean UL CSA); (**D**) vestibular function (horizontal vHIT gain); (**E**) autonomic function. Orange bars are *RFC1*-positive patients. Red circles in (**B**) and (**C**) represent the same patient. SARA, Scale for Assessment and Rating of Ataxia; UL, upper limb; SNAP, sensory nerve action potentials; CSA, cross-sectional area; vHIT, video head impulse test. ^1^As the modified SNAP asymmetry score is categorical and unrankable we include here the mean UL SNAPs, a secondary measure for representing non-length-dependent neuropathy. ^2^vHIT presented is for best side of each patient.

*RFC1*-positive patients were not more likely to have been investigated for ataxia—only one of the five RFC1-positive patients had had an MRI and that was for investigation of possible epilepsy—and the MRI was normal. Nor were they more ataxic on examination than *RFC1* negative (median SARA score 2.60 versus 2.88 (*P* = 0.99) (Fig. 2A); nor were their bedside head impulse test, dynamic visual acuity (>2 lines lost), or VVOR different between the groups. The one patient with gaze-evoked nystagmus and bilateral positive head impulse test was not *RFC1* positive. Similarly, neuropathy scores were indistinguishable between the two groups: (mean ISS score 6.60 versus 5.68, *P* = 0.46, and mean TCNS score 7.4 versus 6.5, *P* = 0.65).

#### Nerve conduction studies

None of the *RFC1*-positive participants had abnormal motor recordings; however, this was also true of 20 of the 37 *RFC1-*negative patients, so it was not significantly different (*P* = 0.07) between the groups. Four of the five *RFC1*-positive patients satisfied the modified SNAP asymmetry score criteria for non-length-dependent sensory neuropathy;^21^ only one *RFC1*-negative patient had this combination of pure sensory findings and a positive modified SNAP asymmetry score. A further four *RFC1-*negative patients had motor involvement but satisfied the modified SNAP asymmetry score criteria, giving a total of five out of 38 *RFC1*-negative patients who were positive; the difference between the groups was statistically significant (*P* = 0.005). The only abnormality on NCS in the fifth *RFC1-*positive patient was bilaterally absent sural sensory responses. The secondary end-points, the ulnar sensory-motor ratio^28^ and mean UL SNAPs (Supplementary Fig. 1, A and B, respectively), were nominally different between groups, but the Camdessanché score^29^ was not (Table 2).

**Table 2:** Comparison between RFC1 gene expansion positive and negative patient groups

	RFC1 positive^a^	RFC1 negative	*P*–value^b^
Primary end-points^c^			
History: chronic cough	5/0	4/32	0.002*
Exam: SARA score	1.5 (2, 3)	3 (0.13, 4.75)	0.99
NCS: pure sensory/motor involvement	5/0	20/17	0.07
NCS: modified SNAP asymmetry score	4/1	5/32	0.005*
US: reduced mean UL cross-sectional area	4/1	2/36	0.0006*
Video oculography: horizontal gain	1.03 (0.84, 1.08)	0.98 (0.92, 1.06)	0.87
History and exam			
Age	62 (62, 64)1	63.5 (55.25, 71.75)	0.69
Sex (female/male)	3/2	15/23	0.63
Cancer	0/5	7/31	1.00
Alcohol (low intake/high intake)	3/2	26/17	1.00
Cough visual analogue score^d^	50 (48.25, 61.25)	43 (26, 56)	0.63
Age at onset of symptoms	53 (49, 58)	57 (49.25, 63.25)	0.44
Asymmetry of numbness	5/0	11/18	0.019**
Positive bedside head impulse test	1/4	7/30	1.00
Positive bedside VVOR	1/4	3/27	0.48
>2 lines change on dynamic VA	1/4	13/17	0.63
Nerve conduction			
Ulnar sensory-motor amplitude ratio	0.0 (0.0–0.14)	1.0 (0.52, 1.8)	0.013**
Mean UL SNAPs	0.36 (0.29, 0.95) µV	10.3 (7.2, 12.9) µV	0.026**
Mean UL SNAPs < 2.0 µV***	4/1	3/33	0.0016**
Combined NCS and clinical			
NCS: Camdessanché score	7.9 (7.9, 7.9)	5.1 (3.1, 6.2)	0.07
NCS: Camdessanché score at least ‘possible’	4/1	9/26	0.031**
Video oculography (vHIT)			
Bilateral impairment (gain)	1/4	3/35	0.40
Bilateral impairment (catch up saccades)	2/3	6/32	0.23
Horizontal gain (better side)	1.1 (1.02, 1.12)	1.05 (1.03, 1.11)	0.78
Bilateral impairment (gain)	0/5	0/29	NA
Autonomic nervous function			
Autonomic symptom score	6 (5, 6)	3 (1, 5)	0.032**
Autonomic scores	21 (18, 22)	8.5 (4, 13.75)	0.034**

Data are presented as those positive for the feature/those negative for the feature.

^a^Quantitative data are reported as median (lower quartile, upper quartile). ^b^When comparing *RFC1*-positive and *RFC1*-negative groups Fisher’s exact test was used for categorical data and Mann–Whitney U-test for quantitative data. ^c^Autonomic testing is not reported (see text). ^d^Of those who reported chronic cough. *We ended up testing six hypotheses as we couldn’t test autonomic testing. Using the Holm–Bonferroni method, all single asterisked parameters are significant. **These results should be considered exploratory. ***Cutoff calculated from ROC score analysis—note sufficient data only available on 41 subjects.

#### Nerve ultrasound

Four of the five participants who were *RFC1* positive had reduced mean UL CSA compared with just two of the 38 *RFC1*-negative patients (*P* = 0.0006) (Fig. 2C). The median amongst *RFC1*-positive and *RFC1*-negative patients was 4.1 (4.0, 4.9) versus 7.5 (6.5, 8.6) mm^2^ (*P* = 0.002). The other *RFC1*-positive participant with normal mean UL CSA and the *RFC1-*positive patient with the second largest CSA (4.9 mm^2^), both had HbA1c > 45 mmol/mol on this occasion, diagnostic of diabetes (having had borderline results in the past).

Ultrasound was able to identify the one *RFC1*-positive participant who didn’t have a non-length-dependent sensory neuropathy; their only NCS abnormality was absent sural sensory responses (completely normal UL sensory responses) (Fig. 2C).

#### Video oculography assessments

One of the two patients with bilateral vestibulopathy on vHIT had the *RFC1* expansion with VOR gains of 0.46 for the left and 0.57 for the right ear, but the other did not. A second expansion-positive patient had slightly low gains although within normal limits (0.84 bilaterally). These two patients both exhibited abnormal catch-up saccades. The remaining three *RFC1*-positive patients had gains that were either in the middle or towards the upper end of the range of the cohort and no significant catch-up saccades (Fig. 2D). As stated above, no patients in either group had low vVVOR gains.

### Autonomic symptoms and testing

#### Autonomic testing

Unfortunately, two of the five affected patients failed the autonomic testing—one had a cough, which made it impossible to perform the Valsalva manoeuvre, and the other had an electrical artefact, making ECG assessments impossible. Two of the remaining patients had moderately severe autonomic features, and one had nearly normal autonomic function (Fig. 2E).

#### Survey of autonomic symptoms

The patients with *RFC1* expansion had a higher median number of autonomic symptoms: 6 (5, 6) versus 3 (1, 5) *P* = 0.032). Similarly, the median symptom impact score was higher at 21 (18, 22) versus 8.5 (4, 13.75) *P* = 0.034). *RFC1*-positive patients more commonly reported gastroparesis, constipation and impaired sweating of the feet, with higher impact scores for each.

#### Blood tests for confounding factors

None of the *RFC1* patients had abnormal thyroid function tests, low vitamin B_12_ or monoclonal gammopathy. The two Sāmoan *RFC1* patients who had the NC_000004.12:g.39348425 AAAAG[n]AAGGG[n] motif expansion satisfied diagnostic criteria for diabetes on the basis of the repeat blood tests in this study, with HbA1c values of 45 and 46 mmol/mol (normal <40). These patients both had low-titre ANA as well. These are the two *RFC1-*positive patients with the largest mean UL CSA results, including the one in the normal range.

Of the 38 patients negative for *RFC1* expansion, a likely cause for their neuropathy was found in 11: one had had chemotherapy, one had an elevated HbA1c in the diabetic range, four had monoclonal antibodies (one of whom also had low vitamin B_12_), another had low vitamin B_12_, three had high titre ANA, and one was found to have a history of nitrous oxide abuse. A further 11 had a possible cause with five having an HbA1c in the pre-diabetic range, five having a past history of heavy alcohol use, and one having both.

#### Predictive features

**Figure 3:**
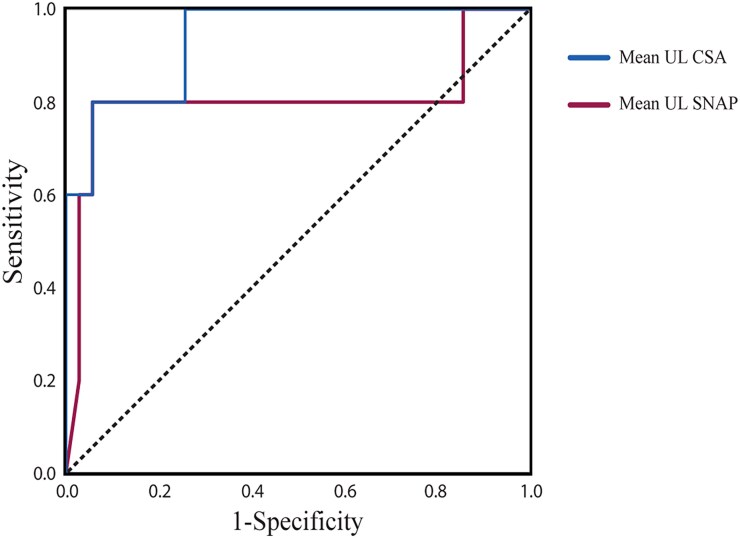
**ROC analysis of mean UL SNAP and CSA.** Mean UL SNAP AUC of 0.808, *P* = 0.037. CSA AUC of 0.937, *P* = 0.001. UL, upper limb; SNAP, sensory nerve action potential; CSA, cross-sectional area.

Neither the presence of ataxia nor bilateral vestibular dysfunction nor autonomic dysfunction predicted the presence of *RFC1* expansion. The presence of a chronic cough on its own was 100% sensitive and 89% specific with a Youden score of 0.89 (Table 3). Receiver Operating Characteristic (ROC) analysis of mean UL CSA showed it provided excellent discrimination (AUC = 0.93, Fig. 3). A mean UL CSA < 5.2 mm^2^ was 80% sensitive and 94% specific with a Youden score of 0.74 (Table 3).

**Table 3:** Reliability of cough, nerve conductions and nerve ultrasound to predict RFC1

	Sensitivity	Specificity	Youden index
Cough	100%	89%	0.89
Mean UL SNAP < 2.0 µV	80%	95%	0.75
Mean UL CSA < 5.2 mm^2^	80%	94%	0.74
Non-length-dependent neuropathy^a^	80%	86%	0.66
Mean UL SNAP < 2.0 µV OR nerve CSA < 5.2 mm^2^	100%	88%	0.88
Non-length-dependent neuropathy OR Mean UL CSA < 5.2 mm^2^	100%	82%	0.82
Cough AND (mean UL SNAP < 2.0 µV OR mean UL CSA < 5.2 mm^2^)	100%	97%	0.97
Cough AND (non-length-dependent neuropathy OR mean UL CSA < 5.2 mm^2^)	100%	97%	0.97

^a^Assessed using modified SNAP asymmetry score.

**Figure 4:**
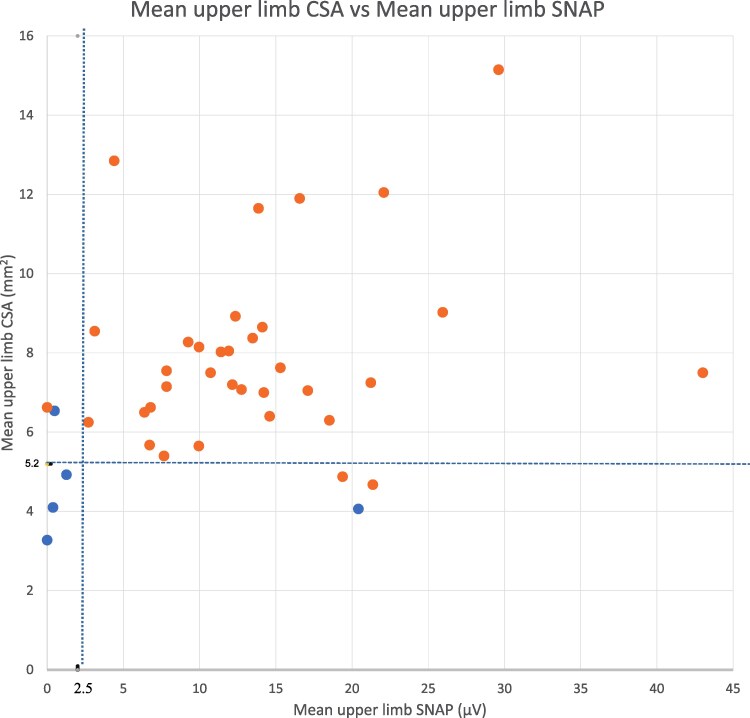
**Combination of Mean UL SNAPs and Mean UL CSAs showing their complementary nature.** Blue points are *RFC1*-positive patients (five patients); brown points show *RFC1-*negative patients (35 patients). *RFC1*-positive patients lie either to the left of the mean UL SNAP = 2.5 line or below the mean UL CSA = 5.2 mm^2^ line with just three false positives. UL, upper limb; SNAP, sensory nerve action potential; CSA, cross-sectional area.

The modified SNAP asymmetry score showed similar sensitivity and specificity (Table 3). However, examination of the data showed that our *RFC1*-positive patients who satisfied these criteria did so based on unilateral or bilateral UL SNAP absence. We found, therefore, that the mean UL SNAP best captured these nerve conduction abnormalities. ROC analysis of mean UL SNAP showed it provided good discrimination (AUC = 0.80, Fig. 3). A mean UL SNAP < 2.0 µV gives 80% sensitivity and 95% specificity with a Youden score of 0.75. Notably, the one *RFC1-*positive patient who didn’t have low SNAPs did have small nerves (Figs 2B and C and Fig. 4). As a result, the combination of (mean UL CSA < 5.2 mm^2^ OR mean UL SNAP < 2.0 µV) gives 100% sensitivity, 88% specificity and a Youden score of 0.88. Further combining cough AND (mean UL CSA < 5.2 mm^2^ OR mean UL SNAP < 2.0 µV) gives an almost perfect discrimination with sensitivity 100%, specificity 97% and a Youden score of 0.97 (Table 3).

## Discussion

This study aimed to systematically assess the clinical features of patients with sensory predominant axonal neuropathy on NCS to determine predictors of patients who carry biallelic pathogenic *RFC1* expansion. We confirm previous findings in similar, larger but less systematic studies^18,19^ that the absence of motor involvement, presence of chronic cough, and a non-length-dependent sensory neuropathy pattern are more common amongst patients with pathological *RFC1* expansions. We also confirm that even if patients are examined thoroughly and prospectively, *RFC1*-positive patients may not show signs of ataxia or vestibulopathy but may be more likely to have autonomic symptoms. Autonomic testing proved technically difficult in this group of patients, so conclusions cannot be drawn in this regard. Importantly, we have demonstrated that small nerves on ultrasound are a feature of *RFC1*-positive patients even when they have otherwise indistinguishable, length-dependent sensory neuropathy. This last finding suggests that the underlying pathology, whatever the nerve conduction pattern, is likely to be a neuronopathy.

### Description of our cohort

While finding predictors of *RFC1* positivity was the main aim of this study, we have also described a new small cohort of patients with *RFC1*.

#### Clinical features

The *RFC1*-positive patients had had neurological symptoms for an average of 9 years. All five participants with *RFC1* expansions had a chronic cough, and this was more common than in *RFC1*-negative patients (*P* = 0.002). This rate is higher than in both previous studies,^18,19^ which may reflect the fact that we asked patients about their cough systematically. Other borderline statistically significant features were asymmetric numbness (*P* = 0.019) and the number and severity of autonomic symptoms on the questionnaire (*P* = 0.013 and *P* = 0.026, respectively). Bedside tests of vestibular dysfunction were present in a minority; their neuropathy signs and symptoms were not particularly marked, and the patients were not ataxic. Our results may be skewed, however, as patients who had more ataxic symptoms on presentation had already been tested for *RFC1* and were excluded from this study.

#### Motor involvement on NCS

None of the *RFC1*-positive participants had motor involvement on NCS testing though this did not differ significantly from *RFC1-*negative patients (*P* = 0.07). The five gene-positive patients make up 20% of those who were found to have a pure sensory axonal neuropathy compared with Curro’s 34%,^18^ and Tagliapietra’s^19^ 53%, the latter is likely to be higher because of different case selection; their study was of patients selected for sural nerve biopsy. Other studies have shown the presence of motor findings on examination, such as weakness, fasciculation or hyperreflexia^36^; however, these studies don’t describe motor NCS in detail.

#### Non-length-dependent neuropathy on NCS

Our study is similar to that of Curro *et al.*^18^ in the proportion of *RFC1*-positive patients who had a non-length-dependent neuropathy—we found 80% compared with their 70% (26% all absent and 46% ‘non-length-dependent’), and this was significantly more common than in gene-negative patients (*P* = 0.005). Our four patients with non-length-dependent neuropathy all satisfied the modified SNAP asymmetry score, the ulnar sensory-motor ratio and the Camdessanché ‘possible neuronopathy’ criteria. Our fifth gene-positive patient had just absent sural SNAPS.

#### Nerve ultrasound findings

Four of the five *RFC1*-positive participants had abnormally small nerves on ultrasound, statistically higher than in *RFC1-*negative patients (*P* = 0.0006). Importantly, this included the one patient with a length-dependent sensory neuropathy on NCS. This supports the concept that in *RFC1*-positive patients, even those with a length-dependent pattern on NCS, the underlying mechanism is likely to be a sensory neuronopathy. This would accord with the known post-mortem findings showing atrophy of dorsal root ganglia.^4^

#### Presence of diabetes

Two of the five *RFC1*-positive participants developed diabetes between their historic NCS and re-testing in this study. Diabetes causes peripheral nerve enlargement on ultrasound.^37-39^ This is important, as both had larger nerves than the other three *RFC1-*positive patients, one within the normal range. It is likely that diabetic enlargement countered the nerve shrinkage due to *RFC1*. Furthermore, it suggests that we should not exclude participants with diabetes when considering *RFC1* as a potential cause of neuropathy, especially in the presence of a cough.

#### Novel RFC1 expansion

Our finding of a novel [AAAAG]_exp_[AAGGG]_exp_ arrangement in two patients is of interest. We reviewed all previous tests in the Pathwest lab, revealing just one other patient with this motif. That patient (known to the investigators) knew of a Māori grandfather but did not know whether they had Sāmoan ancestors. Given that the ancestors of Māori and Cook Islands Māori (part of the Eastern Polynesian peoples) are understood to have migrated east from Western Polynesia (Samoa/Tonga/Tokelau) ∼1000 years ago,^40^ we speculate that the (AAAAG)(n)(AAGGG)(n) allele seen in these two patients may be a precursor of the (AAAGG)(n)(AAGGG)(n) allele seen amongst Māori and Cook Islands Māori.^35^

The exact influence of expansion content and size on phenotype is unclear; although the expansion size of the smaller and larger allele has been shown to have a small effect on age of onset and possibly also on how complex the phenotype is.^41^ Our group^13^ and others^42^ have drawn attention to the complexity of repeat expansions, including hexanucleotide repeats. In other conditions, including Huntington’s disease,^43^ it is not just the length but the structure of the repeats that determines the phenotype. There is a suggestion that the (ACAGG) repeat seen in East Asian and Pacific people might be associated with more motor features,^44^ but otherwise this remains to be explored in *RFC1* expansion patients.

#### Predictive qualities of clinical features

Since sensory predominant axonal neuropathy is relatively common, we sought to identify features of *RFC1*-positive patients’ presentation, which would differentiate those who should have genetic testing. The presence of a chronic cough on its own was quite predictive. However, it is worth noting that in the previous larger studies, it has not been so pervasive as in our cohort.

This study is amongst the first to add peripheral nerve ultrasound to the diagnostic screening for *RFC1*. We have previously shown that patients with CANVAS have smaller peripheral nerves on ultrasound^6,22,23^ and more recently, others have shown that *RFC1*-positive cases have small nerves, regardless of additional CANVAS features.^45^ In keeping with this, small nerves on ultrasound strongly predicted *RFC1* positivity, as did low Mean UL SNAPs. These nerve ultrasound and NCS measures were complementary with *RFC1* patients either having small nerves, low Mean UL SNAPs or both. Combining these features with the presence of cough gives an almost perfect method for finding *RFC1*-positive patients with 100% sensitivity, 97% specificity and a Youden score of 0.97.

Based on these results, we recommend that for patients referred for neurophysiology, the presence of a chronic cough should be enquired about and, if present, should trigger a nerve ultrasound. Small nerves in this situation could justify going directly to *RFC1* genetic testing without needing NCS. Even in the absence of cough, patients with evidence of a non-length-dependent sensory neuropathy, especially if this is characterized by absent UL SNAPs, should also be tested. The presence of other causes such as diabetes shouldn’t preclude this testing. Finally, the finding of a typical length-dependent axonal neuropathy, especially if pure sensory, should be followed up by a nerve ultrasound, and patients with abnormally small nerves should also have *RFC1* genetic testing.

Acquired causes for neuropathy were identified in 10 of the 38 patients with negative *RFC1* testing: this highlights the need for reconsidering the diagnosis where no cause has been found.

The main limitation of this study is that the cohort is relatively small. However, we can have confidence in our findings given how consistent they are with the previous larger studies—in areas that they overlap. Further studies to confirm the new expansion allele amongst Sāmoan patients and to determine how reliably nerve ultrasound detects otherwise unremarkable length-dependent sensory axonal neuropathy will be important.

The strength of our study is our well-defined cohort, with all participants being re-examined by assessors, blinded to other investigators’ findings.

## Conclusion

As the field of CANVAS/*RFC1* spectrum disorder progresses and the broad multi-system phenotype becomes clearer and disease-modifying treatments become available, they are more likely to be aimed at those with milder disease. This will further underline the need to be able to recognize the more limited forms of the condition. Nerve ultrasound is increasingly accessible^46^ and has been touted as complementary to NCS for studying peripheral nerve disease,^47^ especially as a marker for inherited sensory neuronopathy.^48^ This study supports that view.

## Supplementary Material

fcaf434_Supplementary_Data

## Data Availability

The datasets from the current study are available from the corresponding author on reasonable request subject to individual participants’ consent.

## References

[1] Bronstein AM, Mossman S, Luxon LM. The neck-eye reflex in patients with reduced vestibular and optokinetic function. Brain. 1991;114(Pt 1A):1–11. 1998877

[2] Migliaccio AA, Halmagyi GM, McGarvie LA, Cremer PD. Cerebellar ataxia with bilateral vestibulopathy: Description of a syndrome and its characteristic clinical sign. Brain. 2004;127(2):280–293. 14607788 10.1093/brain/awh030

[3] Szmulewicz DJ, Waterston JA, Halmagyi GM, et al Sensory neuropathy as part of the cerebellar ataxia neuropathy vestibular areflexia syndrome. Neurology. 2011;76(22):1903–1910. 21624989 10.1212/WNL.0b013e31821d746e PMC3115806

[4] Szmulewicz DJ, McLean CA, Rodriguez ML, et al Dorsal root ganglionopathy is responsible for the sensory impairment in CANVAS. Neurology. 2014;82(16):1410–1415. 24682971 10.1212/WNL.0000000000000352 PMC4001192

[5] Wu TY, Taylor JM, Kilfoyle DH, et al Autonomic dysfunction is a major feature of cerebellar ataxia, neuropathy, vestibular areflexia “CANVAS” syndrome. Brain. 2014;137(10):2649–2656. 25070514 10.1093/brain/awu196

[6] Pelosi L, Mulroy E, Leadbetter R, et al Peripheral nerves are pathologically small in cerebellar ataxia neuropathy vestibular areflexia syndrome: A controlled ultrasound study. Eur J Neurol. 2018;25(4):659–665. 29316033 10.1111/ene.13563

[7] Cortese A, Simone R, Sullivan R, et al Biallelic expansion of an intronic repeat in RFC1 is a common cause of late-onset ataxia. Nat Genet. 2019;51(4):649–658. 30926972 10.1038/s41588-019-0372-4 PMC6709527

[8] Cortese A, Tozza S, Yau WY, et al Cerebellar ataxia, neuropathy, vestibular areflexia syndrome due to RFC1 repeat expansion. Brain. 2020;143(2):480–490. 32040566 10.1093/brain/awz418 PMC7009469

[9] Aboud Syriani D, Wong D, Andani S, et al Prevalence of RFC1-mediated spinocerebellar ataxia in a North American ataxia cohort. Neurol Genet. 2020;6(3):e440. 32582864 10.1212/NXG.0000000000000440 PMC7274910

[10] Ghorbani F, de Boer-Bergsma J, Verschuuren-Bemelmans CC, et al Prevalence of intronic repeat expansions in RFC1 in Dutch patients with CANVAS and adult-onset ataxia. J Neurol. 2022;269(11):6086–6093. 35864213 10.1007/s00415-022-11275-9 PMC9553829

[11] Montaut S, Diedhiou N, Fahrer P, et al Biallelic RFC1-expansion in a French multicentric sporadic ataxia cohort. J Neurol. 2021;268(9):3337–3343. 33666721 10.1007/s00415-021-10499-5

[12] Traschütz A, Cortese A, Reich S, et al Natural history, phenotypic spectrum, and discriminative features of multisystemic RFC1 disease. Neurology. 2021;96(9):E1369–E1382. 33495376 10.1212/WNL.0000000000011528 PMC8055326

[13] Scriba CK, Stevanovski I, Chintalaphani SR, et al RFC1 in an Australasian neurological disease cohort: Extending the genetic heterogeneity and implications for diagnostics. Brain Commun. 2023;5(4):fcad208. 37621409 10.1093/braincomms/fcad208 PMC10445415

[14] Pellerin D, Wilke C, Traschütz A, et al Intronic FGF14 GAA repeat expansions are a common cause of ataxia syndromes with neuropathy and bilateral vestibulopathy. J Neurol Neurosurg Psychiatry. 2023;95(2):175–179. 10.1136/jnnp-2023-331490 PMC10850669 37399286

[15] Gordon CR, Zaltzman R, Geisinger D, Elyoseph Z, Gimmon Y. Bilateral vestibulopathy as the initial presentation of CANVAS. J Neurol Sci. 2024;460:122990. 38579416 10.1016/j.jns.2024.122990

[16] Hadjivassiliou M, Currò R, Beauchamp N, et al Can CANVAS due to RFC1 biallelic expansions present with pure ataxia? J Neurol Neurosurg Psychiatry. 2023;95(2):171–174. 10.1136/jnnp-2023-331381 PMC10850715 37414537

[17] Van Daele SH, Vermeer S, Van Eesbeeck A, et al Diagnostic yield of testing for RFC1 repeat expansions in patients with unexplained adult-onset cerebellar ataxia. J Neurol Neurosurg Psychiatry. 2020;91(11):1233–1234. 32732387 10.1136/jnnp-2020-323998

[18] Currò R, Salvalaggio A, Tozza S, et al RFC1 expansions are a common cause of idiopathic sensory neuropathy. Brain. 2021;144(5):1542–1550. 33969391 10.1093/brain/awab072 PMC8262986

[19] Tagliapietra M, Cardellini D, Ferrarini M, et al RFC1 AAGGG repeat expansion masquerading as Chronic Idiopathic Axonal Polyneuropathy. J Neurol. 2021;268(11):4280–4290. 33884451 10.1007/s00415-021-10552-3 PMC8505379

[20] Schmitz-Hübsch T, du Montcel ST, Baliko L, et al Scale for the assessment and rating of ataxia. Neurology. 2006;66(11):1717–1720. 16769946 10.1212/01.wnl.0000219042.60538.92

[21] Zis P, Hadjivassiliou M, Sarrigiannis PG, Barker ASJE, Rao DG. Rapid neurophysiological screening for sensory ganglionopathy: A novel approach. Brain Behav. 2017;7(12):e00880. 29299392 10.1002/brb3.880 PMC5745252

[22] Pelosi L, Coraci D, Mulroy E, Leadbetter R, Padua L, Roxburgh R. Ultrasound of peripheral nerves distinguishes inherited sensory neuronopathy of cerebellar ataxia with neuropathy and vestibular areflexia syndrome from inherited axonopathy. Muscle Nerve. 2023;67(1):33–38. 36354069 10.1002/mus.27751

[23] Pelosi L, Leadbetter R, Mulroy E, Chancellor AM, Mossman S, Roxburgh R. Peripheral nerve ultrasound in cerebellar ataxia neuropathy vestibular areflexia syndrome (CANVAS). Muscle Nerve. 2017;56(1):160–162. 27859440 10.1002/mus.25476

[24] Ewing DJ, Clarke BF. Diagnosis and management of diabetic autonomic neuropathy. BMJ. 1982;285(6346):916–918. 6811067 10.1136/bmj.285.6346.916 PMC1500018

[25] Halmagyi GM, Colebatch JG, Curthoys IS. New tests of vestibular function. Baillieres Clin Neurol. 1994;3(3):485–500. 7874404

[26] Halmagyi GM, Curthoys IS. A clinical sign of canal paresis. Arch Neurol. 1988;45(7):737–739. 3390028 10.1001/archneur.1988.00520310043015

[27] MacDougall HG, Weber KP, McGarvie LA, Halmagyi GM, Curthoys IS. The video head impulse test: Diagnostic accuracy in peripheral vestibulopathy. Neurology. 2009;73(14):1134–1141. 19805730 10.1212/WNL.0b013e3181bacf85 PMC2890997

[28] Garcia RU, Ricardo JAG, Horta CA, Garibaldi SG, Nucci A, França MC. Ulnar sensory-motor amplitude ratio: A new tool to differentiate ganglionopathy from polyneuropathy. Arq Neuropsiquiatr. 2013;71(7):465–469. 23857623 10.1590/0004-282X20130063

[29] Camdessanché JP, Jousserand G, Ferraud K, et al The pattern and diagnostic criteria of sensory neuronopathy: A case-control study. Brain. 2009;132(7):1723–1733. 19506068 10.1093/brain/awp136 PMC2702838

[30] Strupp M, Kim JS, Murofushi T, et al Bilateral vestibulopathy: Diagnostic criteria consensus document of the classification committee of the bárány Society1. J Vestib Res. 2017;27(4):177–189. 29081426 10.3233/VES-170619 PMC9249284

[31] Zay Melville I, Yamsuan K, Wu H, Thorne PR, Kobayashi K, Taylor RL. Do measures of gain asymmetry and catch-up saccades improve video head impulse test agreement with caloric results? Clin Neurophysiol Pract. 2024;9:217–226. 39206448 10.1016/j.cnp.2024.07.001 PMC11350461

[32] Rey-Martinez J, Batuecas-Caletrio A, Matiño E, Trinidad-Ruiz G, Altuna X, Perez-Fernandez N. Mathematical methods for measuring the visually enhanced vestibulo–ocular reflex and preliminary results from healthy subjects and patient groups. Front Neurol. 2018;9:69. 29483893 10.3389/fneur.2018.00069 PMC5816338

[33] Zilliox L, Peltier AC, Wren PA, et al Assessing autonomic dysfunction in early diabetic neuropathy. Neurology. 2011;76(12):1099–1105. 21422460 10.1212/WNL.0b013e3182120147 PMC3068012

[34] Holm S . A simple sequentially rejective multiple test procedure. Scand J Stat. 1979;6(2):65–70.

[35] Beecroft SJ, Cortese A, Sullivan R, et al A māori specific RFC1 pathogenic repeat configuration in CANVAS, likely due to a founder allele. Brain. 2020;143(9):2673–2680. 32851396 10.1093/brain/awaa203 PMC7526724

[36] Huin V, Coarelli G, Guemy C, et al Motor neuron pathology in CANVAS due to RFC1 expansions. Brain. 2022;145(6):2121–2132. 34927205 10.1093/brain/awab449

[37] Pitarokoili K, Kerasnoudis A, Behrendt V, et al Facing the diagnostic challenge: Nerve ultrasound in diabetic patients with neuropathic symptoms. Muscle Nerve. 2016;54(1):18–24. 26575030 10.1002/mus.24981

[38] Pelosi L . Nerve ultrasound—A screening tool for diabetic neuropathy. Clin Neurophysiol Pract. 2023;8:113–114. 38152241 10.1016/j.cnp.2023.05.003 PMC10751743

[39] Breiner A, Qrimli M, Ebadi H, et al Peripheral nerve high-resolution ultrasound in diabetes. Muscle Nerve. 2017;55(2):171–178. 27312883 10.1002/mus.25223

[40] Patel SG, Buchanan CM, Mulroy E, et al Potential PINK1 founder effect in Polynesia causing early-onset Parkinson’s disease. Mov Disord. 2021;36(9):2199–2200. 34159639 10.1002/mds.28665

[41] Currò R, Dominik N, Facchini S, et al Role of the repeat expansion size in predicting age of onset and severity in RFC1 disease. Brain. 2024;147(5):1887–1898. 38193360 10.1093/brain/awad436 PMC11068103

[42] Dominik N, Magri S, Currò R, et al Normal and pathogenic variation of *RFC1* repeat expansions: Implications for clinical diagnosis. Brain. 2023;146(12):5060–5069. 37450567 10.1093/brain/awad240 PMC10689911

[43] Dawson J, Kay C, Black HF, et al The frequency and clinical impact of synonymous HTT loss-of-interruption and duplication-of-interruption variants in a diverse HD cohort. Genet Med. 2024;26(11):101239. 39140258 10.1016/j.gim.2024.101239

[44] Scriba CK, Beecroft SJ, Clayton JS, et al A novel RFC1 repeat motif (ACAGG) in two Asia-Pacific CANVAS families. Brain. 2020;143(10):2904–2910. 33103729 10.1093/brain/awaa263 PMC7780484

[45] Salvalaggio A, Cacciavillani M, Tierro B, et al Nerve ultrasound in CANVAS-spectrum disease: Reduced nerve size distinguishes genetically confirmed CANVAS from other axonal polyneuropathies. J Peripher Nerv Syst. 2024;29(4):464–471. 39219417 10.1111/jns.12655

[46] Telleman JA, Grimm A, Goedee S, Visser LH, Zaidman CM. Nerve ultrasound in polyneuropathies. Muscle Nerve. 2018;57(5):716–728. 29205398 10.1002/mus.26029

[47] Preston D, Shapiro B. Electromyography and neuromuscular disorders. Elsevier; 2020.

[48] Pelosi L, van Alfen N. Neuromuscular ultrasound as a marker for inherited sensory neuronopathy. Muscle Nerve. 2023;68(5):718–721. doi:10.1002/mus.27934 37436126

