# Supplementary figures and images for: Nerve ultrasound, neuronopathy and cough predict sensory neuropathy patients with RFC1 expansions
Source: Brain Commun. 2025 Nov 3;7(6):fcaf434. doi: 10.1093/braincomms/fcaf434 (PMC12662233; doi:10.1093/braincomms/fcaf434)

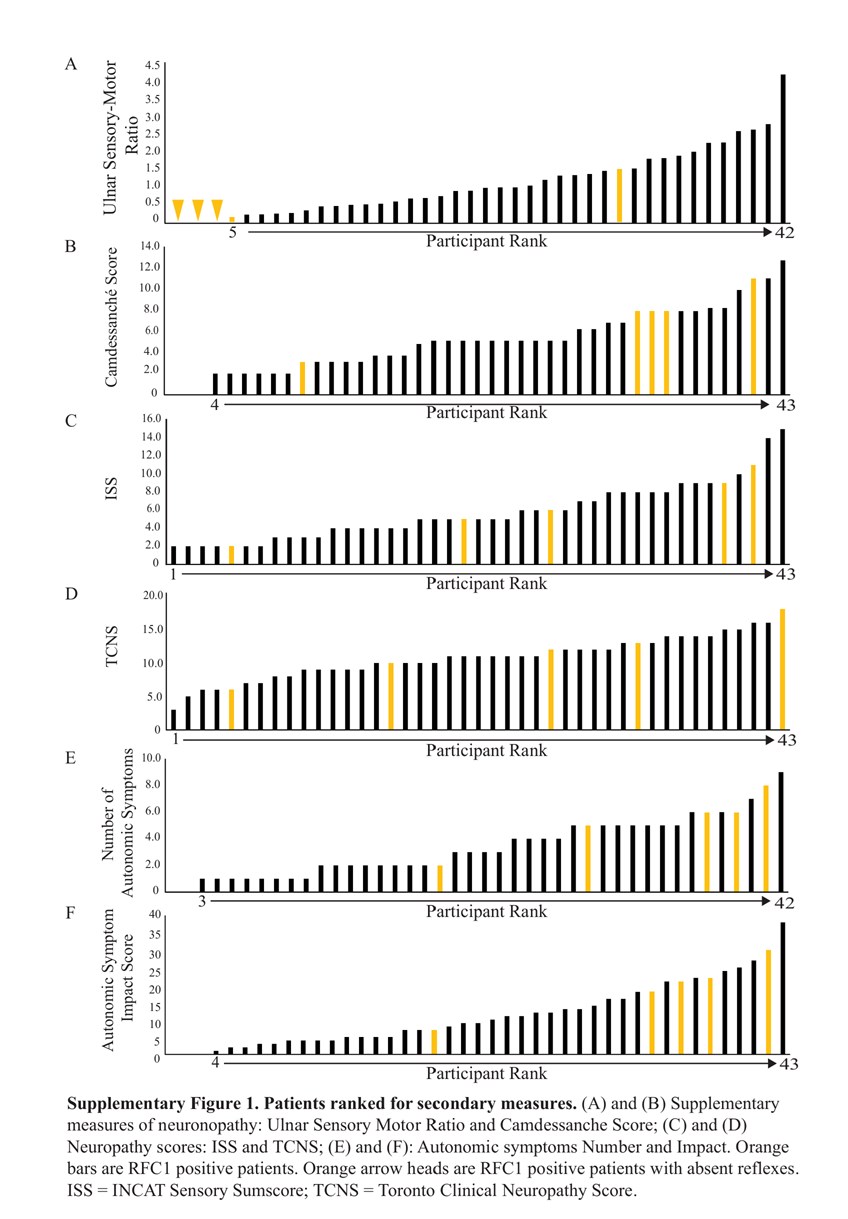

Supplement: fcaf434_Supplementary_Data [file fcaf434_supplementary_data.jpeg]
